# Genetic variations in familial hypercholesterolemia and cascade screening in East Asians

**DOI:** 10.1002/mgg3.520

**Published:** 2018-12-27

**Authors:** Melody Lok‐Yi Chan, Ching‐Lung Cheung, Alan Chun‐Hong Lee, Chun‐Yip Yeung, Chung‐Wah Siu, Jenny Yin‐Yan Leung, Ho‐Kwong Pang, Kathryn Choon‐Beng Tan

**Affiliations:** ^1^ Department of Medicine University of Hong Kong Hong Kong Hong Kong; ^2^ Department of Pharmacology and Pharmacy University of Hong Kong Hong Kong Hong Kong; ^3^ Department of Medicine Ruttonjee Hospital Wan Chai Hong Kong; ^4^ Department of Medicine Pamela Youde Nethersole Eastern Hospital Hong Kong Hong Kong

**Keywords:** *APOB* gene, cascade screening, familial hypercholesterolemia, genetic spectrum, *LDLR* gene

## Abstract

**Background:**

Familial hypercholesterolemia (FH) is a monogenic disorder of lipoprotein metabolism leading to an increased risk of premature cardiovascular disease. Genetic testing for FH is not commonly used in Asian countries. We aimed to define the genetic spectrum of FH in Hong Kong and to test the feasibility of cascade genetic screening.

**Methods:**

Ninety‐six Chinese subjects with a clinical diagnosis of FH were recruited, and family‐based cascade screening incorporating genetic testing results was performed.

**Results:**

Forty‐two distinct mutations were identified in 67% of the index FH cases. The majority of causative mutations were in the *LDLR* gene. The three commonest mutations in the *LDLR* gene were NM_000527.4(*LDLR*): c.1241 T>G, NM_000527.4(*LDLR*): c.1474G>A, and NM_000527.4(*LDLR*): c. 682G>A, and nine novel variants were identified. The NM_000384.2(*APOB*): c.10579 C>T variant of the *APOB* gene was found in 5% of the index subjects. The presence of causative mutation significantly increased the odds of successful family recruitment for screening with an OR of 3.7 (95% CI: 1.53–9.11, *p* = 0.004).

**Conclusion:**

Approximately two‐third of the subjects in this clinically ascertained sample of patients with FH had a discrete genetic basis. Genetic identification improves the response rate and efficiency of family screening.

## 1 INTRODUCTION

Familial Hypercholesterolemia (FH) is the most common inherited disorder of lipid metabolism resulting in high levels of low‐density lipoprotein cholesterol (LDL‐C) and an increased risk of premature atherosclerotic cardiovascular disease (CVD). Early diagnosis and treatment significantly improves the prognosis and reduces the cardiovascular morbidity and mortality. However, FH is underdiagnosed and undertreated in many countries. Active case finding plus family‐based cascade screening has been recommended by national and international bodies to help identify individuals with FH and ensure early intervention (Watts et al., 2014).

The major causative genes underlying monogenic FH are the LDL‐receptor (*LDLR*) gene (OMIM: 606945), the apolipoprotein B‐100 (*APOB*) gene (OMIM: 107730), and/or the proprotein convertase subtilisin/kexin type 9 (*PCSK9*) gene (OMIM: 607786). To date, up to 1,700 different variants have been identified in the *LDLR* gene (Leigh et al., 2017). The spectrum of FH mutations does vary between different countries and ethnic groups, and the available information regarding molecular diagnosis of FH in Asia remains rather limited compared with data in Europe and America. A recent review of the molecular genetics of FH in Han Chinese suggested that the five most frequent mutations were NM_000384.2(*APOB*): c.10579 C>T, NM_000527.4(*LDLR*): c.986G>A, NM_000527.4(*LDLR*): c.1747 C>T, NM_000527.4(*LDLR*): c.1879G>A, and NM_000527.4(*LDLR*): c.268G>A (Chiou & Charng, 2016). Hong Kong has a population of seven million and majority of the population are of Hans descent. Diagnosis of FH in Hong Kong is based on clinical grounds as genetic testing for FH is not available in the public healthcare system. Hence, the aim of this study was to determine the spectrum of genetic mutations in the Chinese subjects with FH in Hong Kong and to test the feasibility of genetic cascade screening.

## 2 MATERIALS AND METHODS

Ethical Compliance: Informed consent was obtained from all subjects, and the study was approved by the Ethics Committee of the University of Hong Kong. Consent to screen family members was obtained from the index case, and invitations to family members were made via the affected family member in view of privacy concerns. Screening was offered to relatives over the age of 18.

Index patients with the clinical diagnosis of FH or severe hypercholesterolemia attending Specialist Clinics from three regional hospitals in Hong Kong were identified from clinic database and invited to participate. A full clinical assessment was performed and secondary causes of hypercholesterolemia were excluded. The diagnosis of FH was retrospectively scored as definite, probable, or possible FH according to the Dutch Lipid Clinic Network (DLCN) Criteria (van Aalst‐Cohen, Jansen, de Jongh, de Sauvage Nolting, & Kastelein, 2004). In 86% of the index patients, pretreatment lipid levels were available in the medical records. For those in whom a pretreatment LDL‐C was not available, an estimate of untreated LDL‐C was obtained using correction factors for statins and ezetimibe treatment (Haralambos, Ashfield‐Watt, & Mcdowell, 2016). Genetic analysis was carried out in these index cases, and their first‐degree relatives (parents, offspring, and siblings) were invited to attend cascade screening to detect potential new cases based on the information from genetic testing (where a family specific causative variant has been identified) or from phenotypic diagnostics. The newly confirmed cases were then taken as new index cases and their first‐degree relatives were subsequently screened whenever possible. Genetic counseling and treatment were offered to individuals diagnosed to have FH after screening. Plasma lipid levels were measured in all family members screened. Blood samples were taken after an overnight fast. Total cholesterol, HDL cholesterol, and triglyceride levels were measured by standardized enzymatic methods, and LDL‐C concentrations were calculated using the Friedewald formula.

For genetic analyses, genomic DNA was isolated from peripheral blood leukocytes using the ReliaPrep^TM^ Blood gDNA Miniprep System (Promega Corporation, Madison, WI, USA). Mutations in the coding regions of *LDLR*,* APOB* exon 22 (codon 1164), 26 (codon 3507 to 3558) and 29 (codons 4396 and 4494), or *PCSK9* genes in the index patients were identified by Sanger sequencing. Standard sequencing was performed using the Applied Biosystems 3730xl DNA Analyzer. The GenBank reference sequences used for *LDLR*,* PCSK9,* and *APOB* were NM_000527.4, NM_174936.3, and NM_000384.2, respectively. Pathogenicity of the identified variants was confirmed through published data in the following reference databases: Leiden Open Variation Database (LOVD) (https://www.LOVD.nl/LDLR)/UCL *LDLR* variant database, Ensembl GRCh38, and ClinVar (https://www.ncbi.nlm.nih.gov/clinvar/), last assessed on 6 April 2018. Pathological assessment of novel variants which were not present in the published database was done using multiple online tools: NCBI Basic Local Alignment Search Tool (BLAST), Sorting Intolerant from Tolerant (SIFT) (Kumar, Henikoff, & Ng, 2009), and Mutation Taster (Schwarz, Cooper, Schuelke, & Seelow, 2014).

## 3 RESULTS

Ninety‐six index patients were recruited, and Table 1 summarizes the clinical profiles and pretreatment lipid levels of the index patients. According to the DLCN criteria, 38 probands had definite FH, 34 probands had probable FH, and 24 had possible FH. All but two subjects were on statins, 57 subjects were on two or more lipid‐lowering agents, and two were receiving plasmapheresis. The on‐treatment plasma total cholesterol level and LDL cholesterol of all index patients were 5.4 ± 1.4 mmol/L and 3.5 ± 1.3 mmol/L, respectively.

**Table 1:** Clinical profiles and pretreatment lipid levels according to the DLCN criteria

DLCN score	Definite FH (score >8)	Probable FH (score 6–8)	Possible FH (score 3–5)
Total (*n*)	38	34	24
Male, *n* (%)	19 (50%)	13 (38%)	11 (46%)
Age at recruitment (years)	51 ± 12	53 ± 10	51 ± 11
Weight (kg)	63.3 ± 10.6	63.7 ± 9.6	65.5 ± 11.5
BMI (kg/m^2^)	24.6 ± 3.4	25.7 ± 3.5	24.5 ± 3.0
CVD, *n* (%)	20 (53%)	17 (50%)	3 (13%)
TC (mmol/L)	11.0 ± 2.3	9.3 ± 1.3	8.1 ± 1.2
TG (mmol/L)	1.5 ± 0.4	1.6 ± 0.7	1.7 ± 0.9
LDL‐C (mmol/L)	9.4 ± 2.1	7.3 ± 1.0	6.0 ± 0.8
HDL‐C (mmol/L)	1.2 ± 0.3	1.4 ± 0.4	1.5 ± 0.4
Tendon xanthomata, *n* (%)	16 (42%)	0	0
Arcus/Xanthelesma, *n* (%)	6 (16%)	1 (3%)	0

*Note*

Values are mean ± *SD*.

Mutations were identified in 67% of the index cases (*n* = 64). Genetic analysis revealed that 54 subjects were heterozygous FH and one subject was a homozygote (Table 2). Nine patients carried two mutant alleles, of which one always affected *LDLR*. In six cases, the second mutation was also in the *LDLR* gene (compound heterozygotes), whereas in three cases the second mutation was in *APOB* gene (double heterozygotes). All the mutations and novel variants, compound and double heterozygous variations identified in this study are shown in Supporting Information Tables S1 and S2, respectively. There are 40 unique mutations in *LDLR* (including nine novel *LDLR* mutations), two unique mutations in *APOB* gene and no gain‐of‐function mutations were identified in *PCSK9* gene. In the *LDLR* gene, the greatest variety of mutations was observed in exon 4, being the largest exon of the *LDLR* gene, with nine different defective loci in 13 FH subjects out of the 96 index cases (14%). The three most common mutations were the NM_000527.4(*LDLR*): c.1241 T>G in exon 9, NM_000527.4(*LDLR*): c.1474G>A in exon 10, and NM_000527.4(*LDLR*): c. 682G>A in exon 4 in this study. Two well‐known variants in *APOB* gene, NM_000384.2(*APOB*): c.10579 C>T, and NM_000384.2(*APOB*): c.10580G>A, both in exon 26, were identified in six index patients (6% of FH subjects), of which three subjects were double heterozygous FH (Supporting Information Table S2).

**Table 2:** Clinical characteristics based on genetic diagnosis

	Heterozygous *LDLR*	Heterozygous *APOB*	Compound/double/homozygous	No mutation
Total (*n*)	51	3	10	32
Definite FH, *n* (%)	26 (51%)	1 (33%)	7 (70%)	4 (13%)
Probable FH, *n* (%)	19 (37%)	1 (33%)	3 (30%)	11 (34%)
Possible FH, *n* (%)	6 (12%)	1 (33%)	0	17 (53%)
Male, *n* (%)	23 (45%)	1 (33%)	5 (50%)	14 (44%)
Age at diagnosis (years)	38 ± 13	37 ± 10	38 ± 12	43 ± 13
CVD, *n* (%)	25 (49%)	0	3 (30%)	12 (38%)
TC (mmol/L)	9.9 ± 1.6	8.8 ± 1.4	11.2 ± 3.3	8.8 ± 1.9
TG (mmol/L)	1.7 ± 1.2	0.8 ± 0.1	1.2 ± 0.6	1.7 ± 0.8
LDL‐C (mmol/L)	8.2 ± 1.6	7.3 ± 1.1	9.8 ± 3.4	6.5 ± 1.5
HDL‐C (mmol/L)	1.3 ± 0.3	1.7 ± 0.5	1.3 ± 0.4	1.5 ± 0.4
Tendon xanthomas, *n* (%)	12 (24%)	0	2 (20%)	2 (6.3%)
Arcus/Xanthelesma, *n* (%)	4 (8%)	1 (33%)	1 (10%)	1 (3%)

*Note*

Values are mean ± *SD*.

The clinical features of the index subjects according to their genetic diagnoses are shown in Table 2. The homozygous FH subject carried the NM_000527.4(*LDLR*): c.1474G>A mutation with a baseline LDL‐C of 9.3 mmol/L. The patient was diagnosed at age 12 with xanthoma. However, he discontinued treatment in his 20s and presented with a myocardial infarction at age 35. One of the compound heterozygotes had interesting variants in intron regions of *LDLR* gene—the pathogenic c.313+5G>A and a frameshift at c.1706‐23 (Supporting Information Table S2). The frameshift mutation was identified in the intron region between *LDLR* exons 11 and 12. However, the shift terminated at c.1706‐23 position before the start of exon 12 without affecting the coding sequences in exon and may not be pathogenic. This patient presented at the age of 21 with a plasma LDL‐C of 9.1 mmol/L. His mother and elder sister also had hypercholesterolemia and were on lipid‐lowering therapies, and there was a strong family history of premature cardiovascular disease. However, segregation analysis could not be performed as his family members were living abroad. Overall, mutation‐positive subjects had significantly higher plasma LDL‐C levels than those without identifiable mutation (*p* < 0.001). Documented clinical evidence of CVD was seen in 44% of mutation‐positive subjects and in 38% of those without identifiable mutation. As expected, subjects with compound or double heterozygosity or homozygosity had higher plasma LDL‐C than those with heterozygous FH (*p* = 0.01).

Genetic cascade family screening was offered to the 64 probands with causative mutations identified. The response rate was 72% and 46 families participated in family screening. A total of 194 first and second‐degree relatives were screened and 101 were identified to have FH. The average number of relatives with FH identified per index case was 2.2. Family screening was also offered to the 32 probands where no causative mutations were found, and the diagnosis of FH in first‐degree relatives was made by phenotypic criteria. Only 13 families attended (response rate 41%) and 38 first‐degree relatives were screened. Nineteen relatives were clinically diagnosed to have FH using phenotypic criteria, and the average number of relatives with FH detected per index case was 1.5. Although the number of relatives with FH identified per index case was fairly similar when diagnosis was made based on genetic or phenotypic criteria, the presence of causative mutation significantly increased the response rate to family screening (OR of 3.7, 95% CI: 1.53–9.11, *p* = 0.004). All newly diagnosed FH subjects received counseling and were started on medical therapy.

To evaluate what was the optimal plasma LDL‐C cutoff level to predict the presence of pathogenic mutation(s) in all subjects screened (including probands and family members), receiver operating characteristic (ROC) curve was generated and the point with the maximum Youden index (*J *= sensitivity + specificity−1) was determined on the ROC curve. The area under ROC curve was 0.90, and the optimal plasma LDL‐C cutoff level obtained using Youden index was 5.5 mmol/L, with a sensitivity of 89% and specificity of 80%. Raising the plasma LDL‐C cutoff level to 6.0 mmol/L increased the specificity to 86% but dropped the sensitivity to 80%.

## 4 DISCUSSION

In this clinically ascertained sample of Chinese patients with FH and/or severe hypercholesterolemia in Hong Kong, we have shown that two‐third of the patients had a discrete genetic basis with the majority of causative mutations in the *LDLR* gene. A recent review identified a total of 143 different *LDLR* mutations in FH subjects of Han Chinese descent (Chiou & Charng, 2016). Majority of the Chinese population in Hong Kong originated from southern China, and the three most common mutations in the *LDLR* gene were the NM_000527.4(*LDLR*): c.1241 T>G (10%), NM_000527.4(*LDLR*): c.1474G>A (6%), and NM_000527.4(*LDLR*): c. 682G>A (4%). The distribution of the common mutations in Hong Kong seems to differ from those reported in China and Taiwan (Chiou & Charng, 2012; Jiang et al., 2015). The common mutations reported in Chinese populations from the North of China (NM_000527.4(*LDLR*): c.1879G>A and NM_000527.4(*LDLR*): c.313+1G>A) and from Taiwan (NM_000527.4(*LDLR*): c.986G>A and NM_000527.4(*LDLR*): c.1747 C>T) were also present in our population but at a lower frequency. The NM_000527.4(*LDLR*): c.1241 T>G mutation, found in 10% of our cohort, is not commonly found in North American and European populations (Abul‐Husn et al., 2016; Khera et al., 2016; Kusters et al., 2011; Wang et al., 2016). Nine novel variants on *LDLR* gene had been identified in our study, and prediction of pathogenicity was based on in silico analysis. Functional studies have not yet been performed to determine the impact of variants/mutations. However, in pedigrees where sufficient family members were available for testing, co‐segregation could be demonstrated. The only homozygous subject in our study carried the second most prevalent *LDLR* mutation NM_000527.4(*LDLR*):c.1474G>A in exon 10. Similar to previous studies, subjects with compound or double heterozygosity, or homozygosity had more severe phenotype (Sjouke et al., 2015). It has been shown that plasma LDL‐C level can be used as an indication to proceed with genetic testing (Civeira et al., 2008). The optimal plasma LDL‐C cutoff level to predict the presence of disease‐causing mutation(s) in our cohort was 5.5 mmol/L which was similar to that reported in Korean population (Shin et al., 2015).

Mutations in the *APOB* gene accounts for approximately 6–10% of all FH cases in European populations (Sharifi, Futema, Nair, & Humphries, 2017), with NM_000384.2(*APOB*): c.10580G>A being the commonest mutation. Likewise, mutations in the *APOB* gene were seen in 6% of our patients with FH with three double heterozygous patients harboring both *APOB* and *LDLR* mutations. In contrast to Caucasian populations, NM_000384.2(*APOB*): c.10579 C>T was the most frequent *APOB* mutation in our study, and this variant was also the main *APOB* gene mutation among FH subjects in Taiwan and in China (Chiou & Charng, 2012; Jiang et al., 2015). We have sequenced all the coding regions of the *PCSK9* gene and did not find any gain‐of‐function mutations in our cohort. Mutations in the *PCSK9* gene are found in <1% of FH subjects in most populations. In a recent large study comprising of several cohorts and case–control studies with over 26,000 participants (Khera et al., 2016), mutations in the *PCSK9* gene was identified in 0.6% of individuals found to harbor a mutation linked to FH. In Japan, the *PCSK9* gain‐of‐function NM_174936.3(*PCSK9*): c.94G>A variant is reported to affect ~6% of heterozygous FH subjects and is possibly more common due to a founder gene effect (Mabuchi et al., 2014).

It has been shown that cascade testing strategies (incorporating genetic testing results when available or LDL‐C when genetic testing is not available) are highly effective means to identify new cases. Genetic cascade screening has been most successful in the Netherlands. The average number of relatives with FH detected per index case was about 8.6 and this was mainly due to the high participation rates among relatives (Umans‐Eckenhausen, Defesche, Sijbrands, Scheerder, & Kastelein, 2001; Wonderling et al., 2004). In our study, we have not provided screening for family members under the age of 18 as ethical approval has not been obtained to cover the screening of children. The yield in the number of relatives identified with FH per index case in our study, around 2.2, was similar to that reported in Norway using genetic cascade screening (Leren et al., 2004). The yield in the number of relatives identified with FH per index case using phenotypic criteria and LDL‐C was 1.5 and was slightly higher than that reported in United Kingdom (around 0.4–0.7) when clinical phenotypic criteria were used in family screening (Hadfield et al., 2009; Marks, Thorogood, Neil, Humphries, & Neil, 2006). Although it has been argued that genetic testing of FH may not be necessary or indeed cost‐effective, our results clearly confirm that one of the potential benefits of genetic testing is in the response rate in cascade family screening. In keeping with studies in other populations, we have found that having an identifiable genetic cause increases the likelihood of family members agreeing to participate in screening (Ademi et al., 2014; Watts et al., 2014). Genetic testing therefore improves the efficiency of cascade family screening and enables patients to be identified at a younger age.

In this study, we have recruited patients mainly with severe phenotypes, and the highly selected nature of our cohort is a limitation. Approximately one‐third of our FH patients did not have an identifiable genetic cause. This is similar to what has been reported in the literature and may be due to a number of reasons. We did not screen for mutations in minor genes, such as *APOE*,* ABCG5*,* ABCG8*,* LIPA*, or *STAP1* which can phenotypically resemble FH (Hegele et al., 2015). Large deletions or insertions in the *LDLR* which may not be readily identified by the conventional Sanger sequencing method may have been missed as we have not employed complementary testing method such as multiplex ligation‐dependent probe amplification (MLPA) (Wang, Ban, & Hegele, 2005). It has been shown that only 4% of FH index cases were carriers of novel large rearrangements in a recent study of clinically suspected FH patients in Singapore (Pek et al., 2018). It is also possible that as yet undiscovered monogenic causes of hypercholesterolemia could explain the phenotype in some of our subjects. Furthermore, subjects with a relatively lower LDL‐C may have a polygenic cause and carry a disproportionately high burden of multiple small effect common variants that raises plasma LDL‐C.

In conclusion, majority of causative mutations in Chinese FH individuals in Hong Kong are in the *LDLR* gene. Three main mutations were NM_000527.4(*LDLR*): c.1241 T>G, NM_000527.4(*LDLR*): c.1474G>A, and NM_000527.4(*LDLR*): c. 682G>A, which together accounted for a combined frequency of 21% among the FH subjects. Genetic cascade screening is feasible and having an identifiable genetic cause increases the willingness of family members to attend for screening.

## CONFLICT OF INTEREST

Authors declare no conflict of interest.
