# Supplementary material for: Genetic variations in familial hypercholesterolemia and cascade screening in East Asians
Source: Mol Genet Genomic Med. 2018 Dec 27;7(2):e00520. doi: 10.1002/mgg3.520 (PMC6393658; doi:10.1002/mgg3.520)
Supplement: Supplementary file 1 [file MGG3-7-na-s001.docx]

**Supplementary Table 1: *In silico* analysis of mutations identified in Hong Kong FH subjects**

| **Mutation** | **Protein change** | **Exon** | **Number of patients** | **dbSNP^b^** |  | **Clinical Significant^c^** |  |
| --- | --- | --- | --- | --- | --- | --- | --- |
|  |  |  |  |  |  |  |  |
| ***APOB*** |  |  |  |  |  |  |  |
| NM_000384.2: c.10579 C>T | p.(Arg3527Trp) | 26 | 5 | rs144467873 |  | Pathogenic |  |
| NM_000384.2: c.10580G>A | p.(Arg3527Gln) | 26 | 1 | rs5742904 |  | Pathogenic |  |
|  |  |  |  |  |  |  |  |
| ***LDLR*** |  |  |  |  | ***LDLR*-LOVD^a^** |  |  |
| NM_000527.4: c.-137 C>T | promoter region |  | 1 | rs879254373 | LDLR_001262 | VUS |  |
| NM_000527.4**:** c.133 G>T | p.(Val45Phe) | 2 | 1 |  | *Novel* | *Predicted Disease-causing* |  |
| NM_000527.4**:** c.313+1 G>A | Splice donor variant | Intron 3-4 | 2 | rs112029328 | LDLR_000045 | Pathogenic |  |
| NM_000527.4**:** c.313+5 G>A | Intron variant | Intron 3-4 | 1 | rs879254467 | LDLR_000892 | Likely pathogenic |  |
| NM_000527.4**:** c.364 A>T | p.(Ile122Phe) | 4 | 1 | rs879254493 | LDLR_000653 | Likely pathogenic |  |
| NM_000527.4**:** c.427 T>A | p.(Cys143Ser) | 4 | 1 |  | *Novel* | *Predicted Diseasing-causing* |  |
| NM_000527.4**:** c.530 C>T | p.(Ser177Leu) | 4 | 1 | rs121908026 | LDLR_001756 | Pathogenic |  |
| NM_000527.4**:** c.550 T>C | p.(Cys184Arg) | 4 | 1 | rs879254572 | LDLR_000077 | Pathogenic |  |
| NM_000527.4**:** c.599 T>G | p.(Phe200Cys) | 4 | 1 | rs879254586 | LDLR_000085 | VUS |  |
| NM_000527.4**:** c.610 T>C | p.(Cys204Arg) | 4 | 1 |  | *Novel* | *Predicted Diseasing-causing* |  |
| NM_000527.4**:** c.681 C>G | p.(Asp227Glu) | 4 | 1 | rs121908028 | LDLR_001815 | Pathogenic |  |
| NM_000527.4**:** c.682 G>T | p.(Glu228Ter) | 4 | 2 | rs121908029 | LDLR_001818 | Pathogenic |  |
| NM_000527.4**:** c.682 G>A | p.(Glu228Lys) | 4 | 4 | rs121908029 | LDLR_000105 | Pathogenic |  |
| NM_000527.4**:** c.769 C>T | p.(Arg257Trp) | 5 | 3 | rs200990725 | LDLR_000115 | VUS |  |
| NM_000527.4**:** c.986 G>A | p.(Cys329Tyr) | 7 | 1 | rs761954844 | LDLR_000150 | Likely pathogenic |  |
| NM_000527.4**:** c.1016 T>C | p.(Leu339Pro) | 7 | 1 |  | *Novel* | *Predicted Disease-causing* |  |
| NM_000527.4**:** c.1019 G>A | p.(Cys340Tyr) | 7 | 1 | rs755757866 | LDLR_000837 | Likely pathogenic |  |
| NM_000527.4**:** c.1048 C>T | p.(Arg350Ter) | 7 | 1 | rs769737896 | LDLR_000147 | Pathogenic |  |
| NM_000527.4**:** c.1055 G>A | p.(Cys352Tyr) | 7 | 1 | rs193922566 | LDLR_001339 | Likely pathogenic |  |
| NM_000527.4**:** c.1073 G>C | p.(Cys358Ser) | 8 | 1 |  | *Novel* | *Predicted Disease-causing* |  |
| NM_000527.4**:** c.1105 G>A | p.(Val369Met) | 8 | 1 | rs730882097 | Not provided | Not provided |  |
| NM_000527.4**:** c.1132 C>T | p.(Gln378Ter) | 8 | 1 | rs879254802 | LDLR_000727 | Pathogenic |  |
| NM_000527.4**:** c.1222 G>A | p.(Glu408Lys) | 9 | 1 | rs137943601 | LDLR_001379 | Likely pathogenic |  |
| NM_000527.4**:** c.1241 T>G | p.(Leu414Arg) | 9 | 10 | rs748554592 | LDLR_000372 | Likely pathogenic |  |
| NM_000527.4**:** c.1246 C>T | p.(Arg416Trp) | 9 | 1 | rs570942190 | LDLR_000180 | Pathogenic |  |
| NM_000527.4**:** c.1258 A>C | p.(Thr420Pro) | 9 | 1 |  | *Novel* | *Predicted Disease-causing* |  |
| NM_000527.4**:** c.1432 G>A | p.(Gly478Arg) | 10 | 2 | rs144614838 | LDLR_000198 | VUS |  |
| NM_000527.4**:** c.1474 G>A | p.(Asp492Asn) | 10 | 6^#^ | rs373646964 | LDLR_000205 | Likely pathogenic |  |
| NM_000527.4**:** c.1525 A>T | | p.(Lys509Ter) | 10 | 1 |  | *Novel* | *Predicted Disease-causing* |
| NM_000527.4**:** c.1567 G>A | | p.(Val523Met) | 10 | 1 | rs28942080 | LDLR_000075 | Pathogenic |
| NM_000527.4**:** c.1606 delT | | p.(Trp536Glyfs*12) | 11 | 1 |  | *Novel* | *Predicted Disease-causing* |
| NM_000527.4**:** c.1706-23 | | Frameshift | Intron 11-12 | 1 |  | *Novel* | *Prediction  Not available* |
| NM_000527.4**:** c.1747 C>T | | p.(His583Tyr) | 12 | 3 | rs730882109 | LDLR_000234 | Pathogenic |
| NM_000527.4**:** c.1765 G>A | | p.(Asp589Asn) | 12 | 3 | rs201971888 | LDLR_000236 | VUS |
| NM_000527.4**:** c.1879 G>A | | p.(Ala627Thr) | 13 | 2 | rs879255066 | LDLR_000259 | Likely pathogenic |
| NM_000527.4**:** c.1957 G>T | | p.(Val653Phe) | 13 | 1 | rs879255085 | LDLR_001114 | VUS |
| NM_000527.4**:** c.2054 C>T | | p.(Pro685Leu) | 14 | 1 | rs28942084 | LDLR_000276 | Pathogenic |
| NM_000527.4**:** c.2072 C>A | | p.(Ser691Ter) | 14 | 1 | rs369943481 | LDLR_001597 | Pathogenic |
| NM_000527.4**:** c.2086 T>G | | p.(Cys696Gly) | 14 | 1 |  | *Novel* | *Predicted Disease-causing* |
| NM_000527.4**:** c.2108-2114 dup | | p.(Arg706Alsfs) | 14 | 1 | rs879255141 | LDLR_000675 | Pathogenic |

| ^a^ Leiden Open Variant Database [https://www.LOVD.nl/LDLR](https://www.lovd.nl/LDLR) ^b^ 1000 Genomes Browser  ^c^ https://www.ncbi.nlm.nih.gov/clinvar/ , Mutation Taster (<http://www.mutationtaster.org/>)  VUS, variant of uncertain significance. | | |  |
| --- | --- | --- | --- |
| ^#^ Homozygous subject included |  |  |  |

**Supplementary Table 2: Mutations detected in compound and double heterozygotes**

|  | First Mutation | | Second Mutation | |  |
| --- | --- | --- | --- | --- | --- |
| Number of subjects | cDNA | Protein change | cDNA | Protein change | *LDLR*-LOVD^a^ |
|  | |  |  |  |  |
| Compound heterozygotes | |  |  |  |  |
| 1 | NM_000527.4**:** c.-137 C>T | promoter region | NM_000527.4**:** c.2054 C>T | p.(Pro685Leu) | LDLR_001262, LDLR_000276 |
| 3 | NM_000527.4**:** c.769 C>T | p.(Arg257Trp) | NM_000527.4**:** c.1765 G>A | p.(Asp589Asn) | LDLR_000115, LDLR_0000236 |
| 1 | NM_000527.4**:** c.313+5 G>A | Intron variant | NM_000527.4**:** c.1706-23 | Intron11-12 | LDLR_000892, Intron* |
| 1 | NM_000527.4**:** c.599 T>G | p.(Phe200Cys) | NM_000527.4**:** c.1132 C>T | p.(Gln378Ter) | LDLR_000085, LDLR_000727 |
|  |  |  |  |  |  |
| Double heterozygotes | |  |  |  |  |
| 1 | NM_000527.4**:** c.1432 G>A | p.(Gly478Arg) | NM_000384.2: c.10579 C>T | p.(Arg3527Trp) | LDLR_000198, rs144467873^b^ |
| 1 | NM_000527.4**:** c.986 G>A | p.(Cys329Tyr) | NM_000384.2: c.10579 C>T | p.(Arg3527Trp) | LDLR_000150, rs144467873^b^ |
| 1 | NM_000527.4**:** c.1567 G>A | p.(Val523Met) | NM_000384.2: c.10579 C>T | p.(Arg3527Trp) | LDLR_000075, rs144467873^b^ |
|  |  |  |  |  |  |

^a^ Leiden Open Variant Database [https://www.LOVD.nl/LDLR](https://www.lovd.nl/LDLR)

^b^ 1000 Genomes Browser

*Frameshift at intron 11-12 until c.1706-23
